# COVID-19 Diagnosis on Chest Radiograph Using Artificial Intelligence

**DOI:** 10.7759/cureus.31897

**Published:** 2022-11-26

**Authors:** Dhiraj Baruah, Louis Runge, Richard H Jones, Heather R Collins, Ismail M Kabakus, Morgan P McBee

**Affiliations:** 1 Radiodiagnosis, Medical University of South Carolina, Charleston, USA; 2 Radiology, Medical University of South Carolina, Charleston, USA; 3 Pediatric Radiology, Medical University of South Carolina, Charleston, USA

**Keywords:** receiver operating characteristic (roc) analysis, rt-pcr, chest radiography, covid, artificial intelligence

## Abstract

Background: The coronavirus disease 2019 (COVID-19) pandemic has disrupted the world since 2019, causing significant morbidity and mortality in developed and developing countries alike. Although substantial resources have been diverted to developing diagnostic, preventative, and treatment measures, disparities in the availability and efficacy of these tools vary across countries. We seek to assess the ability of commercial artificial intelligence (AI) technology to diagnose COVID-19 by analyzing chest radiographs.

Materials and methods: Chest radiographs taken from symptomatic patients within two days of polymerase chain reaction (PCR) tests were assessed for COVID-19 infection by board-certified radiologists and commercially available AI software. Sixty patients with negative and 60 with positive COVID reverse transcription-polymerase chain reaction (RT-PCR) tests were chosen. Results were compared against results of the PCR test for accuracy and statistically analyzed by receiver operating characteristic (ROC) curves along with area under the curve (AUC) values.

Results: A total of 120 chest radiographs (60 positive and 60 negative RT-PCR tests) radiographs were analyzed. The AI software performed significantly better than chance (p = 0.001) and did not differ significantly from the radiologist ROC curve (p = 0.78).

Conclusion: Commercially available AI software was not inferior compared with trained radiologists in accurately identifying COVID-19 cases by analyzing radiographs. While RT-PCR testing remains the standard, current advances in AI help correctly analyze chest radiographs to diagnose COVID-19 infection.

## Introduction

Coronavirus disease 2019 (COVID-19) is caused by severe acute respiratory syndrome coronavirus-2 (SARS-CoV-2) virus. Multiple types of coronavirus infection were described before, and COVID-19 is the seventh in that group [1]. The severe acute respiratory syndrome (SARS) outbreak in China in 2002 was caused by SARS-CoV-1. Middle East respiratory syndrome (MERS) in Jordan in 2012 was also due to coronavirus. The initial case of COVID-19 was reported in Wuhan, Hubei Province of China on December 31, 2019, and the World Health Organization (WHO) announced SARS-CoV-2 infection as a pandemic in March 2020. The incubation period of this viral infection is between 2-14 days and symptoms start between 8-16 days [1]. Till now a significant number of people died because of this infection worldwide. Clinical examination, laboratory investigation, and imaging all play important roles in diagnosing COVID-19 infection and evaluating the need for hospital admission [2,3]. RT-PCR (real-time reverse transcription-polymerase chain reaction) viral nucleic acid is the gold standard method for diagnosis of COVID-19. However, this test can be false negative in a significant percentage of cases and an alternate test will be contributory.

Chest radiography is a commonly performed investigation in patients with COVID-19 and correct diagnosis may be achieved with classic imaging appearance. Abnormal findings in COVID-19 positive cases commonly include consolidation and ground glass opacity, most often located in the lower lung fields, peripherally and appearing bilaterally [4]. Beyond diagnosis, the severity of chest radiographs upon admission has been shown to provide a predictive value of the disease course [5].

Although chest radiographs are available in most of the world, RT-PCR testing may not be available, and AI (artificial intelligence) software may be helpful to diagnose those cases where there is a limitation for chest radiologists.

The concept of AI is not a novel one, but limitations in computing power have restricted its utility until recently. Original AI developments were governed by algorithms with predefined rules that guided utility for a certain, single task [6]. While these methods can be trained for and successful at assisting in the role for which they were designed, they are unable to evolve with additional information, limiting their adaptive utility. To harness the power of increasingly available computing power, the next generation of AI has been established using deep learning (DL), which allows for continuous enhancement of problem-solving by learning from data as it is interpreted [6]. In medicine and radiology, the most common DL implementation is through convolutional neural networks (CNN), a strategy that employs successive layers to interpret data, further refining the reasoning and output as more layers are added [7].

Radiology presents unique opportunities and challenges for the implementation of DL AI. Currently, different uses are defined by the detection of disease, classification, and segmentation [7,8]. Concisely, detection consists of identifying abnormal features on imaging and is widely employed in thoracic imaging to assist in identifying pulmonary nodules [6,7]. The classification includes sorting images into one or more from a series of options, aiding to stratify tumors [6,7]. Segmentation takes advantage of AI’s ability to recognize patterns in changes in density to separate images into components, for example, separating overlying organs or separating tumorous and non-tumorous tissue [6,7]. Beyond diagnostic and therapeutic usage, AI has the potential to streamline workflow processes, including screening and prioritization of new cases and prediction of observer fatigue [9].

In this study, we aim to compare commercially available AI software with trained radiologists in diagnosing COVID-19 infection via chest radiograph interpretation.

## Materials and methods

This is an IRB (Institutional Review Board) approached retrospective study (BlueDocAI PILOT, IRB number Pro00105121 and BlueDocAI PILOT with control group, IRB number Pro00107373). COVID-19 RT-PCR positive and negative cases were selected from April 2020 to October 2020. Inclusion criteria were - adults more than 18 years old, RT-PCR report, and chest radiograph within two days of the RT-PCR test (all patients undergone the same type of RT-PCR test). Sixty radiographs from COVID-19 RT-PCR positive patients and 60 radiographs from COVID-19 RT-PCR negative patients were chosen. Commercially available cloud-based software (BlueDocAI) was used to evaluate those radiographs. Three board-certified radiologists (with 17, 6, and 6 years of experience after fellowship) evaluated those radiographs, and the presence or absence of COVID-19 findings was noted.

**Figure 1:**
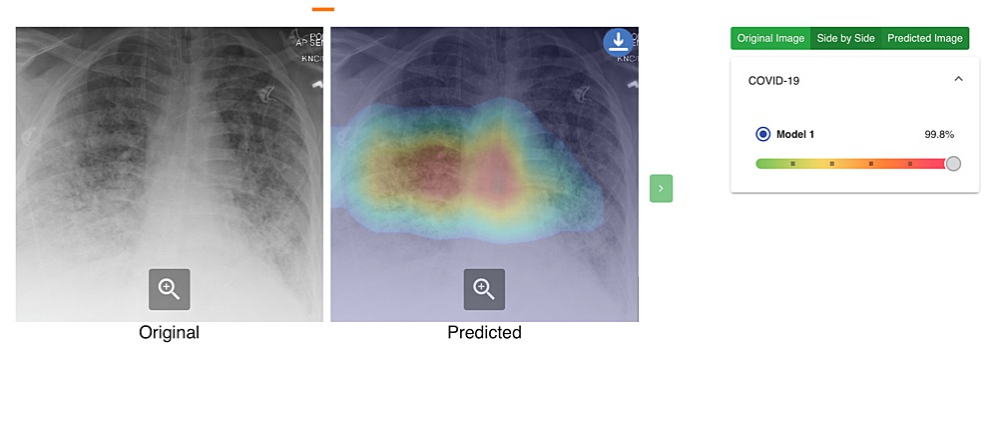
Chest Radiograph with AI Evaluation A 54-year-old female presented with cough, sore throat, and weakness to the emergency department with a positive COVID test. Plain radiograph showing bilateral lower lobe predominant airspace and interstitial opacities. Radiologists diagnosed this as positive for COVID from the chest radiograph and the AI software calculated a 99.8% prediction of COVID.

The AI software output included a percentage score for each patient, with a higher percentage indicating a greater likelihood of a positive result (Figure 1). Findings from the radiologists and the software were compared statistically. Differences between positive and negative cases in age and AI algorithm percentage were evaluated with Mann-Whitney U tests and were described with medians (Mdn), median absolute deviations (MADs), and ranges. Receiver operating characteristic (ROC) curves were characterized with area under the curve (AUC) values along with 95% confidence intervals. Differences between AI and radiologist ROC AUCs were evaluated with z-tests. Two-sided p-values are reported, and statistical significance was set at the α < .05 threshold. Analyses were conducted with SPSS version 27 (IBM: Armonk, NY).

## Results

**Figure 2:**
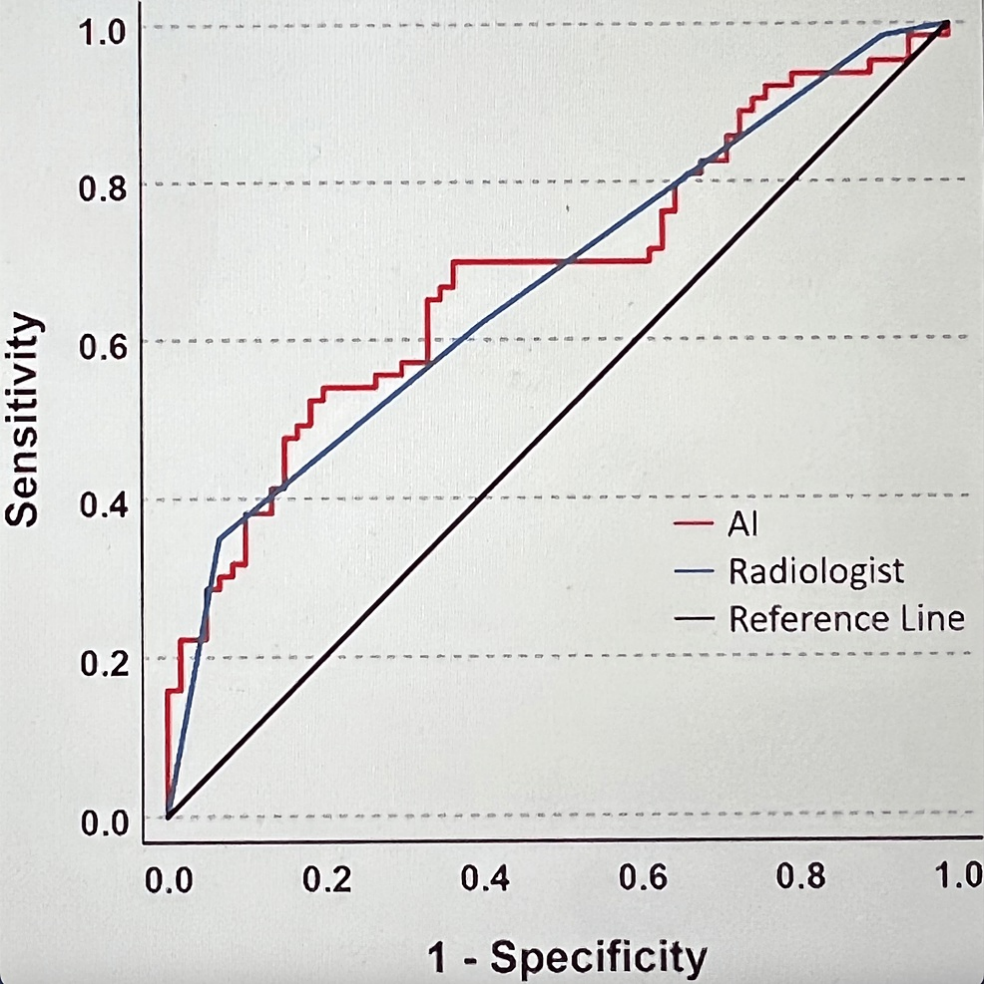
Receiver Operating Curve COVID evaluations by radiologists (AUC = 0.67, 95% CI = 0.57-0.76) and the AI algorithm (AUC = 0.68, 95% CI = 0.59-0.78) yielded AUC values that were significantly better than chance, p = 0.001 and p < 0.01, respectively. These curves are very similar and did not differ significantly from one another, p = 0.78. AI: artificial intelligence, AUC: area under the curve, CI: confidence interval

COVID evaluations by radiologists (AUC = 0.67, 95% CI = 0.57-0.76) and the AI algorithm (AUC = 0.68, 95% CI = 0.59-0.78) yielded AUC values that were significantly better than chance, p = 0.001 and p < 0.01, respectively (Figure 2). These curves are very similar and did not differ significantly from one another, p = 0.78. The AI algorithm correctly identified 65.1% of positive cases and 65.0% of negative cases. The radiologists correctly identified 60.9% of positive cases and 60.0% of negative cases (Table 1). The AI algorithm yielded significantly higher percentages of likely COVID-19 diagnosis for positive patients (Mdn = 73.70%, MAD = 21.90%, range: 0.90%-100.00%) than for negative patients (Mdn = 53.85%, MAD = 15.95%, range: 3.20%-96.8%), p < 0 .001.

**Table 1:** Sensitivity and Specificity of AI vs Radiologist in Diagnosing COVID from Chest X-ray The AI algorithm correctly identified 65.1% of positive cases and 65.0% of negative cases. The radiologists correctly identified 60.9% of positive cases and 60.0% of negative cases. AI: artificial intelligence

Method	Sensitivity	Specificity
AI	65.1%	65.0%
Radiologist	60.9%	60.0%

## Discussion

AI is not new and was initially described in a workshop held at Dartmouth College in 1956 and the concept relies on computer learning from experience [10]. The use of AI in image analysis is a rapidly growing field in radiology research and already has many clinical applications. AI techniques recognize images as data and evaluate complex patterns in imaging data to provide quantitative information [6]. The significant number of available digital data to train algorithms with high-power computational methods are helping to create AI models that now can match and even surpass human beings in task-specific applications [11-14].

Our study shows that this commercially available cloud-based software is helpful in diagnosing COVID-19 infection from chest radiographs. Recent studies have reported similar results, including supplementary use of AI software to assist physician diagnosis of COVID-19 using chest radiograph, where AI assistance increases sensitivity from 47% to 61% in one study and increases the precision of the radiologist findings from 65.9% to 81.9% in another [15,16]. Other studies have also reported comparable success rates between AI and trained radiologist diagnosis of COVID-19 through CT and chest radiography interpretation [17-19]. Additionally, recent studies have reported comparable sensitivities and specificities to our results, measuring 41.6% and 60% in one study, respectively, and 55% and 83% in the first round of another which took serial images in patients, improving to a sensitivity of 79% and a specificity of 70% on subsequent readings [19,20]. Studies have also shown the importance of applying AI in imaging (both chest radiography and CT) for diagnosing and prognosticating COVID-19 infection [21-27].

Applying these results, AI may be important in monitoring and prognosticating COVID-19 patients and can play a complementary role in a busy clinical practice. This AI technique can help with challenges of timely diagnosis and appropriate management including quarantine or contact tracing. AI is proven to be helpful in many areas of radiology including the diagnosis of cancer.

Our study has limitations and one important is the retrospective nature of the evaluation. Prospective randomized control studies based on this will help in the future to validate this technique, however, initial results are promising. Single-center evaluation by trained radiologists is of advantage compared and similarity of reporting style. However, we reached a consensus in some cases where the interpretation was not similar at the initial evaluation.

## Conclusions

Appropriate use of AI software can help in radiology, particularly in situations where trained individuals may not be available to interpret those imaging. This cloud-based commercially available software showed similar performance to trained academic radiologists evaluating findings of COVID-19 infection in chest radiographs.
